# ITRAQ-based quantitative proteomic analysis of processed *Euphorbia lathyris* L. for reducing the intestinal toxicity

**DOI:** 10.1186/s12953-018-0136-6

**Published:** 2018-04-17

**Authors:** Yu Zhang, Yingzi Wang, Shaojing Li, Xiuting Zhang, Wenhua Li, Shengxiu Luo, Zhenyang Sun, Ruijie Nie

**Affiliations:** 1 College of Traditional Chinese Pharmacy, Beijing University of Chinese Medicine, North Third Ring Road, Number 11, Chaoyang District, Beijing, 100029 People’s Republic of China; 2 Institute of Chinese Materia Medica, China Academy of Chinese Medical Sciences, Dong cheng District, Dongzhimen Neixiang Street on the 16th, Beijing, 100700 People’s Republic of China

**Keywords:** *Euphorbia lathyris*, Proteomics, iTRAQ, Bio-pathway

## Abstract

**Background:**

*Euphorbia lathyris* L., a Traditional Chinese medicine (TCM), is commonly used for the treatment of hydropsy, ascites, constipation, amenorrhea, and scabies. Semen Euphorbiae Pulveratum, which is another type of ***Euphorbia lathyris*** that is commonly used in TCM practice and is obtained by removing the oil from the seed that is called paozhi, has been known to ease diarrhea. Whereas, the mechanisms of reducing intestinal toxicity have not been clearly investigated yet.

**Methods:**

In this study, the isobaric tags for relative and absolute quantitation (iTRAQ) in combination with the liquid chromatography-tandem mass spectrometry (LC-MS/MS) proteomic method was applied to investigate the effects of *Euphorbia lathyris* L. on the protein expression involved in intestinal metabolism, in order to illustrate the potential attenuated mechanism of *Euphorbia lathyris* L. processing. Differentially expressed proteins (DEPs) in the intestine after treated with Semen Euphorbiae (SE), Semen Euphorbiae Pulveratum (SEP) and Euphorbiae Factor 1 (EFL_1_) were identified. The bioinformatics analysis including GO analysis, pathway analysis, and network analysis were done to analyze the key metabolic pathways underlying the attenuation mechanism through protein network in diarrhea. Western blot were performed to validate selected protein and the related pathways.

**Results:**

A number of differentially expressed proteins that may be associated with intestinal inflammation were identified. They mainly constituted by part of the cell. The expression sites of them located within cells and organelles. G protein and Eph/Ephrin signal pathway were controlled jointly by SEP and SE. After processing, the extraction of SEP were mainly reflected in the process of cytoskeleton, glycolysis and gluconeogenesis.

**Conclusions:**

These findings suggest that SE induced an inflammatory response, and activated the Interleukin signaling pathway, such as the Ang/Tie 2 and JAK2/ STAT signaling pathways, which may eventually contribute to injury result from intestinal inflammatory, while SEP could alleviate this injury by down-regulating STAT1 and activating Ang-4 that might reduce the inflammatory response. Our results demonstrated the importance of Ang-4 and STAT1 expression, which are the target proteins in the attenuated of SE after processing based on proteomic investigation. Thus iTRAQ might be a novel candidate method to study scientific connotation of hypothesis that the attenuated of SE after processing expressed lower toxicity from cellular levels.

## Background

*Euphorbia lathyris* L. is an effective but toxic traditional Chinese medicine (TCM) derived from the family of euphorbiaceae. It can expel water retention with drastic purgative effects, namely, breaking up the static blood and eliminating masses and is often used for the treatment of hydropsy, ascites, anuresis and constipation, amenorrhea, scabies [1, 2]. It shows several side effects such as irritation and inflammation intense on the skin, mouth and gastrointestinal tract irritation, carcinogenic, and so on. The gastrointestinal mucosa irritation mainly manifested as serious diarrhea. Traditionally, Semen Euphorbiae Pulveratum (SEP), which is another type of *Euphorbia lathyris* L., is commonly used in TCM practice and is obtained by removing the oil from the seed which is called paozhi. After processing, the toxicity and the capacity of diarrhea was decreased obviously [3]. Interestingly, considerable research efforts have been devoted to the studies on the effect of SEP and SE on diarrhea. Whereas, the intestine protein changes related to intestinal toxicity and the main mechanisms of reducing toxicity by processing of SE remain poorly understood.

With the improvement of two-dimensional polyacrylamide gel electrophoresis (2D-PAGE) and mass spectrometry [4], considerable research efforts have been devoted to the application of proteomics to find possible involved signals in toxic injure induced by some toxins or to determine the modes of action and mechanisms involved in drug- or chemical-induced toxicity [5, 6]. The isobaric tags for relative and absolute quantitation (iTRAQ) technique is one of the most widely used, innovative and common quantitative proteomics approaches that measure the qualitative and quantitative changes in protein content of a cell or tissue in response to treatment or disease and determine protein-protein and protein-ligand interactions [7]. It can simultaneously analyze 4–8 different specimens, thus increasing throughput while reducing experimental error [8, 9]. iTRAQ labeling coupled with LC-MS/MS is sensitive, automated, and multidimensional and can detect large molecules (> 20 kDa) [10]. ITRAQ is suitable for exploratory studies of the processing mechanisms.

In our study, we applied iTRAQ approach to processing for *Euphorbia lathyris*-induced intestinal toxicity and to identify candidate biomarkers for main mechanisms underlying processing of SE. Bioinformatics analysis including GO analysis, pathway analysis, and network analysis were done to find possible differential pathways. Additionally, the investigation suggested that Euphorbiae factor 1(EFL_1_), isolated from *Euphorbia lathyris*, is the main and active diterpenoids which might mediate diarrhea [11]. We also demonstrated EFL_1_ group to further compare the DEPs induced by SE and SEP. Finally, western blot analysis was applied further to identify candidate biomarkers, and to confirm and validate significance of the proteomic findings. These results provided a first insight into scientific connotation of hypothesis that the attenuated of SE after processing expressed lower toxicity from cellular levels in mice model and described an efficient method for mechanisms of toxic TCM processing.

## Methods

### Samples

#### Experimental animals

KM mice (SPF grade, 18–22 g) were purchased from Sibeifu Co., Ltd. (Beijing, China). All experiments were approved by the Animal Care Committee. Mice were kept at room temperature (23 ± 1 °C) and 55 ± 5% humidity. All experiments were conducted in accordance with the Guiding Principles for the Care and Use of Laboratory Animal, as adopted by the Committee on Animal Research at Beijing University of Chinese Medicine.

#### Extracts preparations of semen euphorbiae and semen euphorbiae Pulveratum

Pieces of Euphorbiae Semen (batch number, 1203070692; origin, Jiangxi province, China) were purchased from Anhui Bozhou HuQiao Chinese Herbal Pieces plant. Petroleum ether extract of Semen Euphorbiae, petroleum ether extract of Semen Euphorbiae Pulveratum was provided by Shandong University of Traditional Chinese Medicine. The extraction and isolation methods of Semen Euphorbiae had been published in these articles [12, 13]. Euphorbiae factor 1 was isolated from the petroleum ether extracts of semen Euphorbia by our team [13, 14].

### Proteomics extraction procedures

#### Protein preparation

After 12 h of fasting, KM mice were randomly divided into 4 groups (*n* = 10 for each group): the group 1 was served as a control, and received only blank 1% sodium carboxymethyl cellulose solution; meanwhile group 2 was the extracts of SE and group 3 was the extraction of SEP, in which the mice were orally administered at the dose of 1.5 ml/20 g and 1.0 ml/20 g, respectively, with the same amount of crude drug. In order to validate the results induced by SE and SEP, group 4 was administered 20 mg/20 g Euphorbiae factor 1(EFL_1_) to further verify the protein networks. Mice then received standard diet and water ad libitum. 6 h later, mice were sacrificed, from which the colon were obtained and frozen in liquid nitrogen immediately until they were used for analysis.

#### Protein isolation

The colon tissue samples were ground into powder in liquid nitrogen, extracted with Lysis buffer (7 M urea (Bio-Rad, 161–0731), 2 M Thiourea (Sigma-Aldrich, T7875), 4% CHAPS (Bio-Rad, 161–0460)) containing complete protease inhibitor Cocktai (Roche, 04693116001). The cell was lysed by sonication at 200 W for 60s and then extracted 30 min at room temperature, centrifuged at 4 °C, 15000 g for 20 min. Before the protein processing, each 5 individual protein samples were mixed equally into 1 specimen. As a result of the strategy, each group contained 2 sample pools, and these sample pools were enrolled to be conducted in subsequent experiments*.*

#### Bradford analysis

Total protein concentration of the samples was determined using a Bradford Assay [15]. Standards of BSA were prepared and all samples and standards were analyzed in duplicate. Protein concentrations and standards of BSA were determined at 595 nm on an Multiskan MK3 UV–vis spectrophotometer (Thermo, U.S.) with 10 μL sample reacted with 300 μL Thermo Scientific Pierce Coomassie Plus Bradford Assay (Part No. 23238) 20 min.

#### Protein reduction, alkylation, and digestion

Filter-aided sample preparation (FASP) method was used to digest protein based on Jacek R Wis’niewski et al. [16]. The 200 μg calculated protein samples were added to centrifuge tube and 25 mM DTT was added and the samples were incubated at 60 °C for 1 h. Samples were incubated for 10 min in the dark after adding 50 mM IAA at room temperature and then centrifuged at 12,000 rpm for 20 min using Ultrafiltration centrifugal tube(NWCO:10 K). 100 μL Dissolution Buffer(iTRAQ ® Kit Dissolution Buffer, AB Sciex, USA, PN:4381664) was added to the filter and centrifuged at 12,000 rpm for 20 min. This step was repeated three times.50 μL trypsin, totally 4 μg, was added and samples were incubated at 37 °C overnight. After trypsin digestion, samples were centrifuged at 12,000 rpm for 20 min, the digested peptides were collected at the bottom of the tube and mixed with 50 μL Dissolution Buffer. Finally 100 μL samples were obtained.

#### iTRAQ labeling

Each iTRAQ reagent tube (tags-113-121) had 150 μl isopropanol added and vortexed thoroughly, then centrifuged. 50 μl samples (equal to 100 μg digested peptides) were transferred to new tubs and processed according to the manufacturer’s protocol for 8-plex iTRAQ reagent (AB Sciex, PN:4390812) by incubation at RT for 2 h with gentle shaking. The labeled peptide mixtures were then pooled and dried by vacuum centrifugation. Samples were labeled respectively with different isobaric tags as follow: EFL_1_ samples labeled 113 and 114, control samples labeled 115 and 116, and extraction of SE samples labeled 117 and 118, extraction of SEP samples labeled 119 and 121. The peptides were labeled with the isobaric tags, incubated at room temperature for 2 h. The labeled peptide mixtures were then pooled and dried by vacuum centrifugation.

#### iTRAQ-labeled peptide fractionation and proteomic analysis by LC-MS/MS

The iTRAQ-labeled peptide mixtures were re-suspended in buffer A (2% ACN, pH 10) and centrifuged at 14,000 g for 20 min. High pH reversed-phase chromatography was performed to separate the trypsin digestion peptide. The supernatant was loaded onto a 4.6 × 250 mm Durashell-C_18_ containing 5-μm particles. The peptides were eluted at a flow rate of 0.7 mL/min with a 51-min gradient:0-10 min,5.0% B (Mobile phaseA:2%ACN,98%ddH_2_O,pH 10;Mobile phaseB:98%ACN,2%ddH_2_O,pH 10);10–13.4 min,5%-8.%B;13.4–31.7 min,8.5%–20.5%B;31.7-41 min,20.5%–31.0%B; 41-46 min,31%–90%B;46-47 min,90.0–95.0%B;47-48 min, 95%–5%B;48-51 min,5%B. The eluted peptides were obtained 40 fractions and finally pooled into 10 fractions through Peak shape.

Then the fraction was re-suspended in 20 μL buffer A (2% ACN, 0.1% FA)and centrifuged at 12,000 rpm for 10 min and 10 μL supernatant was loaded onto a 12 cm × 75 μm EASY-Spray column (C_18_,3 μm). The samples were loaded at 300 nL/min with mobile phase A: 100% dd H_2_O/0.1% Formic acid; mobile phase B: 100% ACN/0.1%FA. The gradient as follows:0-13 min,5–8%B;13-90 min,8030%B;90-100 min,30–50%B;100-105 min,50–95%B;105-115 min,95%B;115-116 min,95–5%B;116-126 min,5%B.

The peptides were subjected to Nano-electrospray ionization followed by mass spectrometry (MS/MS) using a Q-Exactive mass spectrometer (Thermo Scientific) coupled with an online micro flow HPLC system. Key parameter settings for the Thermo Q-Exactive mass spectrometer were as follows:

spray voltage floating (ISVF) 2.3KV, Capillary Temperature:320 °C, Ion source: EASY-Spray source, declustering potential (DP) 100 V.

Full MS:Resolution:70000FWHM;Full Scan AGC target:3e6;Full Scan Max.IT:20 ms;Scan range:300-1800 m/z;

dd-MS2:Resolution:17500 FWHM;AGC target:1e5;Maximum IT:120 ms;Intensity threshold:8.30E + 03;Fragmentation Methods:HCD;NCE:32%;Top N:20.

### Bioinformatics analysis

Annotations of identified proteins were done with GO for biological processes, molecular functions and cellular component. The analysis were carried out using the Database for Annotation Visualization and Integrated Discovery. Tagged samples were normalized by comparing median protein ratios for the reference channel. Protein quantitative ratios were calculated from the median of all peptide ratios. The proteins with a relative expression of > 1.32 or < 0.68, and with a *P*-value < 0.05 selected as statistically significance to ensure up- and downregulation authenticity. The selection parameter was based on the overrepresented GO terms with gene enrichment analysis of *p* < 0.05. The protein lists were further analyzed by UniProt database (http://www.uniprot.org/uniprot/?query=taxonomy:10090) which gave all canonical pathways, interactions, and network construction with significant enrichment of the input proteins based on data from the UniProt Database, Biocarta, etc. [17]

### Western blot analysis

Western blot analysis were performed to confirm the presence of differentially expressed proteins. Colons from mouse were washed with ice-cold saline and triturated under Liquid Nitrogen. 200 mg powder were lysed in 1.5 ml RIPA buffer and incubated on ice for 60 min, sonicated for 60s, followed by centrifugation at 12,000×g for 15 min at 4 °C. The total protein concentration was measured using the BCA protein assay kit (Applygen Technologies Inc. Beijing, China). The supernatant lysates were diluted in 5× SDS sample buffer and boiled for 5–10 min.

Proteins from individual samples were separated on SDS-PAGE gels and transferred electrophoretically onto PVDF membranes (Millipore, Billerica, MA, USA). The membranes were blocked for 2 h at room temperature with 3% non-fat dried milk in Tris-buffered saline (TBST, 20 mM Tris-HCl, 137 mM NaCl, and 0.1% Tween 20, pH 7.6). Then, the membranes were incubated overnight at 4 °C in a primary antibody against Anti-STAT1 antibody(Abcam, USA), Rabbit Anti-Angiopoietin 4(Beijing Biosynthesis Biotechnology Co., Ltd.,China), Rabbit and Mouse Anti-β-actin(ZS-Bio. Co., Ltd. Beijing, China). The membranes were then washed with TTBS three times and incubated with horseradish peroxidase-conjugated secondary antibodies (ZS-Bio. Co., Ltd. Beijing, China), Peroxidase-Conjugated Goat anti-Mouse IgG (H + L) (ZB-2305) and Peroxidase-Conjugated Goat anti-Rabbit IgG (H + L) (ZB-2301).Proteins were detected using an enhanced chemiluminescence (ECL) method (Super ECL plus Detection Reagent, Applygen Technologies Inc.P1010). Protein bands were imaged using a ChemiScope 3300 Mini bio-imaging system (Clinx Science Instruments Co., Ltd. (CSI), Shanghai, China). Bands were normalized with β- actin as an internal control. Protein expressions were quantified by chemi analysis and Ang4 and STAT1 were normalized to the beta-actin of each sample. These experiments were each conducted five times.

## Results and discussion

### Protein profiling

**Fig. 1:**
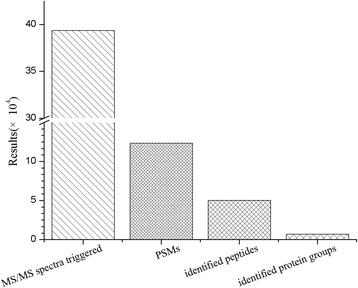
Basic information statistics of proteome by iTRAQ. MS/MS spectra are the secondary mass spectrums, and PSMs are the secondary mass spectrums after quality control. Protein is identified by Proteome Discoverer 1.4 software

MS raw data files were converted into MGF files using Proteome Discoverer 1.4 (PD 1.4, Thermo), and the MGF data files were searched by using the Mascot search engine (Matrix Science, London, UK; version 2.3.02) to identify proteins. Each confident protein identification involves at least one unique peptide. For protein quantification, it was required that a protein contained at least two unique spectra. The quantitative protein ratios were weighted and normalized by the median ratio in Mascot. As shown in Fig. 1, a total of 393,357 MS/MS spectra which are the secondary mass spectrums were identified by iTRAQ-coupled 2D LC-MS/MS analysis in mice intestine tissues. Among them, 123,136 peptide spectrum-match (PSM) were found. In addition, the LC-MS/MS analysis employed here resulted in identification of 50,007 total peptides with 6727 identified protein groups.

### Identification of differentially expressed proteins using iTRAQ labeling and LC-MS/MS

Through analysis with software, data were processed using the Proteome Discoverer Software 4.0 utilizing the Mascot (Matrix Science,London, U.K.; version 2.3.0) Algorithm. In this algorithm, Parameters set for the searching were iTRAQ eight plex peptide-labeled, trypsin digestion with only two maximum miss cleavage, carboxymate for cysteine residue and oxidation for methionine. The tolerances were specified as ±15 ppm for peptides and ± 20 mmu for MS/MS fragments. The mice protein database was downloaded from UniProt. The false discovery rate (FDR) was controlled at the 1% level. Distributional normality and homogeneity of variance were tested for numerical data. Values were given as mean ± SD. To reduce probability of false peptide identification, only peptides with a fold change cut-off ratio of > 1.32 or < 0.68 and ones with *p*-values smaller than 0.05 in the analysis (where *P*-value < 0.05 indicates > 95% confidence of a change in protein concentration irrespective of the magnitude of the change) was selected to designate differentially expressed proteins. The similar experimental design was described in previous study [18–20]. Among them, proteins that displayed significantly altered expression levels comparing with the control group were considered as up-regulated or down-regulated differentially expressed proteins (DEPs), respectively. With this filter, we identified 103 DEPs in EFL_1_ group, including 82 up-regulated proteins and 21 down-regulated proteins. Besides, regarding to 70 DEPs from SE-treated group compared to control group, 47 proteins were up-regulated, and 23 proteins were down-regulated. Moreover, there were 96 up-regulated proteins and 26 down-regulated proteins, totaling 122 proteins in the SEP-treated groups were identified relative to control. Further analysis indicated that the three test groups shared 7 DEPs in the colon tissues of mice after intersection, of which, 5 proteins were down-regulated and 2 proteins up-regulated (Table 1). Meanwhile, there were 295 differentially expressed proteins in the colon tissues of mice in union of DEPs of SE and SEP, EFL_1_, of which, 70 proteins were down-regulated and 225 proteins up-regulated (Table 2). These proteins were subjected to gene-ontology enrichment.

**Table 1:** Related information of differentially expressed protein (DEPs) by iTRAQ analysis after intersection

Acc no. (NCBI)	Prot names	Gene names	Control	SE	SEP	EFL_1_
Down-regulated proteins						
Q3TMQ6	Angiogenin-4	Ang4	1	0.5795	0.6082	0.549
Q62010	Oviduct-specific glycoprotein	Ovgp1	1	0.4252	0.5825	0.451
Q80ZA0	Intelectin-1b (Intelectin-2)	Itln1b	1	0.4847	0.6715	0.498
Q8R1M8	Mucosal pentraxin	Mptx1	1	0.5352	0.5652	0.559
V9GXU2	C2 domain-containing protein 3	C2cd3	1	0.5372	0.636	0.463
Up-regulated proteins						
F6R782	IQ domain-containing protein E	Iqce	1	3.496	4.4437	4.691
Q9D1X0	Nucleolar protein 3 (Apoptosis repressor with CARD)	Nol3 Arc	1	1.3665	1.5167	1.345

*Acc no* Accession number, *Prot name* Protein name, *SE* Semen Euphorbiae, *SEP* Semen Euphorbiae Pulveratum, *EFL*_*1*_ Euphorbiae Factor 1

**Table 2:** Summary table showing significantly up-regulated or down-regulated proteins identified by iTRAQ Analysis after combine together

Acc no.(NCBI)	Pro names	Gene names	Control	SE	EFL_1_	SEP
Up-regulated						
Q62010	Oviduct-specific glycoprotein	Ovgp1 Chit5 Ogp	1	0.4252	0.451	0.5825
A2BDX4	Potassium voltage-gated channel subfamily G member 1	Kcng1	1	0.4347	0.856	0.6645
P97816	Protein S100-G	S100 g Calb3 S100d	1	0.4485	0.599	0.653
Q8BV14	Ankyrin repeat domain-containing protein 55	Ankrd55	1	0.4652	0.636	/
Q80ZA0	Intelectin-1b (Intelectin-2)	Itln1b Itln2 Itlnb	1	0.4847	0.498	0.6715
Q8R1M8	Mucosal pentraxin	Mptx1 Mptx	1	0.5352	0.559	0.5652
V9GXU2	C2 domain-containing protein 3	C2cd3	1	0.5372	0.463	0.636
P07146	Anionic trypsin-2	Prss2 Try2	1	0.5465	0.776	0.5967
D6RFD6	Protein RFT1 homolog	Rft1	1	0.5687	0.771	4.6342
Q8VCV1	Alpha/beta hydrolase domain-containing protein 17C	Abhd17c	1	0.5707	1.271	0.7095
Q3TMQ6	Angiogenin-4	Ang4	1	0.5795	0.549	0.6082
Q08189	Protein-glutamine gamma-glutamyltransferase E	Tgm3 Tgase3	1	0.6085	0.728	0.528
Q8CIM3	D-2-hydroxyglutarate dehydrogenase, mitochondrial	D2hgdh	1	0.61	0.704	1.0195
Q9D7Z6	Calcium-activated chloride channel regulator 1	Clca1	1	0.649	0.709	0.637
O88273	Gremlin-2 (Protein related to DAN and cerberus)	Prdc	1	0.6542	0.906	1.7397
D6RFQ5	p53 and DNA damage-regulated protein 1	Pdrg1	1	0.6585	0.683	0.6567
Q8BYF6	Sodium-coupled monocarboxylate transporter 1	Slc5a8 Smct Smct1	1	0.6667	0.972	0.767
H3BLD0	ATP synthase mitochondrial F1 complex assembly factor 1	Atpaf1	1	0.6687	0.841	0.9637
Q8BXQ3	Leucine-rich repeat and transmembrane domain-containing protein 1	Lrtm1	1	0.6702	0.982	0.573
A0A075B5L8	Protein Igkv4–79	Igkv4–79	1	0.6722	0.688	0.8432
Q3V341	Protein kinase C zeta type	Prkcz	1	0.6775	0.606	1.052
O88310	Intelectin-1a	Itln1	1	0.6782	0.696	0.7225
Q9D2X6	Colon SVA-like protein	Sval1 mcsp mCG_17084	1	0.6782	0.912	0.5127
Q64339	Ubiquitin-like protein ISG15	Isg15 G1p2 Ucrp	1	0.6887	0.922	0.6737
Q810Q5	Normal mucosa of esophagus-specific gene 1 protein	Nmes1	1	0.693	0.832	0.5877
P21550	Beta-enolase	Eno3 Eno-3	1	0.6965	0.876	0.6672
P56392	Cytochrome c oxidase subunit 7A1, mitochondrial	Cox7a1	1	0.7257	0.755	0.655
P30275	Creatine kinase U-type, mitochondrial	Ckmt1	1	0.7492	0.851	0.6657
Q6T707	Protein Scd4 (Stearoyl-CoA desaturase-4)	Scd4	1	0.768	1.808	1.1152
Q9NYQ2	Hydroxyacid oxidase 2 (HAOX2)	Hao2 Hao3 Haox2	1	0.771	0.721	0.658
P09036	Serine protease inhibitor Kazal-type 3	Spink3	1	0.7765	0.987	0.595
P98086	Complement C1q subcomponent subunit A	C1qa	1	0.785	0.406	0.8317
F8VPP8	Protein Zc3h7b	Zc3h7b	1	0.7887	0.677	0.787
Q5RI75–2	Ras and EF-hand domain-containing protein homolog	Rasef	1	0.7892	0.651	0.6965
A2AGQ3	MAP kinase-activating death domain protein	Madd	1	0.7932	1.327	1.398
E9QNL5	Sulfotransferase	Sult1a1	1	0.796	0.659	0.7287
P00329	Alcohol dehydrogenase 1	Adh1 Adh-1	1	0.7992	1.036	0.5625
Q3UZZ6	Sulfotransferase 1 family member D1	Sult1d1 St1d1	1	0.81	0.632	0.7565
B2RT41	Protein Zfc3h1	Zfc3h1 Ccdc131	1	0.831	0.921	0.6362
P57774	Pro-neuropeptide Y [Cleaved into: Neuropeptide Y	Npy	1	0.835	1.436	1.1532
Q3UW68	Calpain-13 (Calcium-activated neutral proteinase 13)	Capn13 Gm943	1	0.838	0.987	0.669
P13634	Carbonic anhydrase 1	Ca1 Car1	1	0.8425	0.622	0.818
Q9WUG6	Insulin-like peptide INSL5 (Insulin-like peptide 5)	Insl5 Rif Rif2 Zins3	1	0.861	1.429	0.6775
F7BQ76	MPN domain-containing protein (Fragment)	Mpnd	1	0.8617	0.603	1.577
P56393	Cytochrome c oxidase subunit 7B, mitochondrial	Cox7b	1	0.8755	1.075	0.6255
Q80WK2	Organic solute transporter subunit beta	Slc51b Ostb	1	0.881	1.373	1.177
A2A6K0	Troponin I, fast skeletal muscle	Tnni2	1	0.886	0.374	0.965
Q7TPR4	Alpha-actinin-1 (Alpha-actinin cytoskeletal isoform)	Actn1	1	0.888	0.857	0.6745
G3X940	Histone acetyltransferase	Kat6a Myst3	1	0.8887	1.618	1.1427
P01796	Ig heavy chain V-III region A4	0	1	0.8935	1.481	1.0095
G3UVW7	Protein Zfp40 (Zinc finger protein 40)	Zfp40 mCG_13522	1	0.9052	1.53	1.0887
Q9EPS2	Peptide YY	Pyy	1	0.9135	1.349	0.974
G3XA21	MCG134445, isoform CRA_a (Protein Mroh1)	Mroh1 Heatr7a	1	0.922	1.114	1.3435
Q9Z179	SHC SH2 domain-binding protein 1	Shcbp1 Pal	1	0.9295	1.107	1.4725
I6L974	TBC1 domain family member 17	Tbc1d17	1	0.9315	1.155	1.3645
P01631	Ig kappa chain V-II region 26–10	0	1	0.9387	1.75	0.821
P01878	Ig alpha chain C region	0	1	0.942	1.332	0.8282
P57776–2	Elongation factor 1-delta (EF-1-delta)	Eef1d	1	0.9477	0.898	0.6252
D3Z6J0	HemK methyltransferase family member 2, isoform CRA_b	N6amt1 Hemk2 mCG_130002	1	0.9562	1.524	1.4077
Q9WUH1	Transmembrane protein 115 (Protein PL6 homolog)	Tmem115 Pl6	1	0.962	1.161	1.4085
Q8R1U2	Cell growth regulator with EF hand domain protein 1	Cgref1 Cgr11	1	0.9635	0.931	1.4472
A0A087WNJ2	Deleted.	0	1	0.974	0.641	0.7125
E0CYM0	Protein 1700019G17Rik	1700019G17Rik	1	0.9752	1.376	1.0687
D3Z7B5	Protein C330027C09Rik	C330027C09Rik	1	0.978	1.336	1.1042
D3Z652	Testis-expressed sequence 35 protein	Tex35	1	0.9797	0.993	1.3665
F8VQE9	ANK repeat and PH domain-containing protein 3	Agap3	1	0.9855	1.026	1.6535
O88665	Bromodomain-containing protein 7	Brd7 Bp75	1	0.9895	0.928	1.5765
E9Q933	Transmembrane protein 11, mitochondrial	Tmem11	1	0.9942	1.5	1.1595
down-regulated						
6NXH9	Keratin, type II cytoskeletal 73	Krt73 Kb36	1	14.265	1.559	1.4102
F6R782	IQ domain-containing protein E	Iqce	1	3.496	4.691	4.4437
A0A075B6A3	Protein Igha	Igha	1	2.7217	1.208	1.9125
P00687	Alpha-amylase 1	Amy1	1	2.5575	4.341	3.1215
Q8C804	Spindle and centriole-associated protein 1	Spice1 Ccdc52	1	2.3742	1.928	1.8472
O88273	Formin-2	Fmn2	1	2.2107	2.234	3.8712
D3Z1G3	Multiple coagulation factor deficiency protein 2 homolog	Mcfd2	1	2.2085	1.694	1.931
A2AHB7	Potassium channel subfamily T member 1	Kcnt1	1	2.181	1.35	5.51
G3UZX8	Probable JmjC domain-containing histone demethylation protein 2C	Jmjd1c	1	2.1745	1.124	3.0692
P35991	Tyrosine-protein kinase BTK	Btk Bpk	1	2.1057	1.302	1.5725
P70213	Friend virus susceptibility protein 1	Fv1	1	1.847	1.207	1.5947
A0A075B664	Protein Iglv2	Iglv2	1	1.8257	3.016	1.2922
E9Q9F6–2	Cation channel sperm-associated protein subunit delta	Catsperd Tmem146	1	1.7907	1.132	0.6605
P57791	CAAX prenyl protease 2	Rce1 Face2 Rce1a	1	1.6772	1.103	1.4677
Q9QZU9	Ubiquitin/ISG15-conjugating enzyme E2 L6	Ube2l6 Ubce8	1	1.648	3.026	2.0062
A2AF82	Activator of 90 kDa heat shock protein ATPase homolog 2	Ahsa2	1	1.6057	1.363	1.5
F8VQM0	Alkaline phosphatase	Akp3	1	1.6022	1.282	2.631
P11034	Mast cell protease 1	Mcpt1	1	1.602	1.704	1.5607
Q6ZWN5	40S ribosomal protein S9	Rps9	1	1.5622	1.053	1.3207
Q9DBB8	Trans-1,2-dihydrobenzene-1,2-diol dehydrogenase	Dhdh	1	1.5585	1.268	1.6725
Q6NZQ2	DEAD/H (Asp-Glu-Ala-Asp/His) box polypeptide 31	Ddx31	1	1.5305	1.169	1.3397
G5E8C3	G protein-coupled receptor, family C, group 5, member A	Gprc5a mCG_22262	1	1.5077	1.168	1.4337
Q91WP6	Serine protease inhibitor A3N	Serpina3n Spi2	1	1.502	0.995	1.2225
A2A3U8	LON peptidase N-terminal domain and RING finger protein 3	Lonrf3	1	1.5017	1.274	1.9312
P07759	Serine protease inhibitor A3K	Spi2	1	1.4825	0.804	1.2742
Q9DCG2–2	CD302 antigen	Cd302 Clec13a	1	1.469	1.116	1.786
P27005	Protein S100-A8 (Calgranulin-A)	S100a8 Caga Mrp8	1	1.4637	1.522	1.154
P04227	H-2 class II histocompatibility antigen, A-Q alpha chain	H2-Aa	1	1.4617	1.382	0.916
Q8C6B9	Active regulator of SIRT1	Rps19bp1 Aros	1	1.4555	1.094	1.78
P70412	CUB and zona pellucida-like domain-containing protein 1	Cuzd1 Itmap1	1	1.4365	1.325	1.5315
Q9D083–3	Kinetochore protein Spc24	Spc24 Spbc24	1	1.4297	1.978	2.0805
P62984	Ubiquitin-60S ribosomal protein L40	Uba52 Ubcep2	1	1.4247	1.336	1.143
P12804	Fibroleukin	Fgl2 Fiblp	1	1.4215	1.407	1.7527
J3QPY0	Protein 1600014C10Rik	1600014C10Rik	1	1.4165	1.485	1.8247
B1AXR3	Perilipin-2	Plin2	1	1.414	0.975	1.3562
Q9ESG9	Membrane-associated tyrosine- and threonine-specific cdc2-inhibitory kinase	Pkmyt1 Myt1	1	1.4137	1.332	1.9545
P07758	Alpha-1-antitrypsin 1–1 (AAT)	Serpina1a Dom1 Spi1–1	1	1.4085	0.908	1.1527
Q8C7E9	Cleavage stimulation factor subunit 2 tau variant	Cstf2t Kiaa0689	1	1.401	1.082	1.014
F6ZQQ3	26S proteasome non-ATPase regulatory subunit 13	Psmd13	1	1.3935	1.417	2.6332
Q91XL1	Leucine-rich HEV glycoprotein (Protein Lrg1)	Lrg1 Lrg lrhg	1	1.3932	0.949	1.327
Q03145	Ephrin type-A receptor 2	Epha2 Eck Myk2	1	1.3932	1.186	1.522
Q9QXA1	Cysteine and histidine-rich protein 1	Cyhr1 Chrp	1	1.3902	1.191	1.0515
Q8BHZ4	Zinc finger protein 592 (Zfp-592)	Znf592 Kiaa0211	1	1.3865	1.338	1.3052
P07724	Serum albumin	Alb Alb-1 Alb1	1	1.3842	0.816	1.2217
V9GX06	Protein Gm11214	Gm11214	1	1.3835	1.098	1.3607
P29699	Alpha-2-HS-glycoprotein (Countertrypin)	Ahsg Fetua	1	1.382	0.774	1.1715
P14148	60S ribosomal protein L7	Rpl7	1	1.3705	0.953	1.1725
P42232	Signal transducer and activator of transcription 5B	Stat5b	1	1.3705	1.627	1.311
P35980	60S ribosomal protein L18	Rpl18	1	1.3695	0.963	1.176
Q9D1X0	Nucleolar protein 3 (Apoptosis repressor with CARD)	Nol3 Arc	1	1.3665	1.345	1.5167
G3X8Z1	Calcium-activated chloride channel regulator 4A	mCG_119588	1	1.366	1.008	1.3725
P01741	Ig heavy chain V region (Anti-arsonate antibody)	0	1	1.3647	3.709	1.06
A0A087WQ94	Protein Tns1	Tns1	1	1.3562	1.117	0.9982
A2AAC0	Chymotrypsin-C	Ctrc	1	1.354	1.062	1.3185
E9Q8K5	Titin	Ttn	1	1.3532	0.744	1.6037
Q3U3Q1–2	Serine/threonine-protein kinase ULK3	Ulk3	1	1.353	1.188	1.574
Q91YU8	Suppressor of SWI4 1 homolog	Ppan Ssf1	1	1.3522	1.167	1.1937
Q6LC96	RXR alpha 2 (RXR alpha 3)	Rxra RXR alpha	1	1.329	0.984	1.2152
Q3UPV6	Voltage-gated potassium channel subunit beta-2	Kcnab2	1	1.328	1.568	1.183
P62301	40S ribosomal protein S13	Rps13	1	1.3275	1.093	1.1617
P22599	Alpha-1-antitrypsin 1–2 (AAT) (Alpha-1 protease inhibitor 2)	Serpina1b Aat2	1	1.326	0.849	1.1235
Q9EP52	Twisted gastrulation protein homolog 1	Twsg1 Tsg	1	1.3242	1.197	0.9917
E9PV04	Protein Gm8994	Gm8994 Gm5576	1	1.3237	1.14	1.2215
P15119	Mast cell protease 2	Mcpt2	1	1.322	1.36	1.0482
Q3ZAR9	Nr2c2 protein (Nuclear receptor subfamily 2 group C member 2)	Nr2c2	1	1.3202	1.395	1.158
Q8BSI6	R3H and coiled-coil domain-containing protein 1	R3hcc1	1	1.319	1.279	1.569
Q32M21–2	Gasdermin-A2	Gsdma2 Gsdm2	1	1.3125	1.482	1.233
Q80TL0	Protein phosphatase 1E	Ppm1e Camkn	1	1.3082	0.645	1.1805
F6RUC3	Ribonucleoside-diphosphate reductase subunit M2 (Fragment)	Rrm2	1	1.3075	1.238	1.4467
A2ALH2	Putative tRNA	Ftsj1	1	1.296	1.3	1.5377
Q8BGS0–2	Protein MAK16 homolog (Protein RBM13)	Mak16 Rbm13	1	1.2927	1.195	1.334
Q8BHY2	Nucleolar complex protein 4 homolog (NOC4 protein homolog)	Noc4l	1	1.2877	1.455	1.5922
Q99J23	GH3 domain-containing protein	Ghdc D11lgp1e	1	1.287	1.231	1.3732
O35640	Annexin A8	Anxa8 Anx8	1	1.277	1.55	1.1167
Q60590	Alpha-1-acid glycoprotein 1	Orm1 Agp1 Orm-1	1	1.263	1.116	1.4175
P35461	Lymphocyte antigen 6G (Ly-6G)	Ly6g	1	1.2495	0.915	1.331
P42225	Signal transducer and activator of transcription 1	Stat1	1	1.2437	1.533	0.9722
Q8VEJ4	Notchless protein homolog 1	Nle1	1	1.2432	1.251	1.3997
F6S522	Claspin	Clspn	1	1.2415	1.134	7.6765
Q8BHN5	RNA-binding protein 45	Rbm45 Drb1 Drbp1	1	1.2387	1.232	1.4235
P31725	Protein S100-A9	S100a9	1	1.2345	1.351	1.028
F8WJ43	Merlin	Nf2	1	1.234	1.168	1.441
Q8C3X8	Lipase maturation factor 2	Lmf2 Tmem112b Tmem153	1	1.2307	0.928	1.5145
E9Q8D0	Protein Dnajc21	Dnajc21	1	1.227	1.476	1.1372
Q9QXA1–2	Cysteine and histidine-rich protein 1	Cyhr1 Chrp	1	1.2205	1.374	1.054
Q3UW98	Chloride channel calcium activated 7	Clca4b AI747448	1	1.2187	1.401	1.0597
A0A075B5M8	Protein Igkv12–38	Igkv12–38	1	1.218	1.337	1.2272
Q4QRL3	Coiled-coil domain-containing protein 88B	Ccdc88b Ccdc88	1	1.2172	1.485	1.392
Q3TBT3–3	Stimulator of interferon genes protein (mSTING)	Tmem173 Eris Mita	1	1.2167	1.441	1.1297
P08905	Lysozyme C-2 (EC 3.2.1.17) (1,4-beta-N-acetylmuramidase C) (Lysozyme C type M)	Lyz2 Lyz Lyzs	1	1.2162	1.353	1.0305
Q9DCS1	Transmembrane protein 176A (Gene signature 188) (Kidney-expressed gene 2 protein)	Tmem176a Gs188 Keg2	1	1.2157	1.248	1.5587
P84228	Histone H3.2	Hist1h3b	1	1.214	0.511	1.0842
D3Z408	High affinity cGMP-specific 3′,5′-cyclic phosphodiesterase 9A	Pde9a	1	1.2137	1.307	1.3392
E9Q4G7	Casein kinase I isoform alpha	Csnk1a1	1	1.2105	1.47	1.643
P05533	Lymphocyte antigen 6A-2/6E-1 (Ly-6A.2/Ly-6E.1) (Stem cell antigen 1) (SCA-1) (T-cell-activating protein) (TAP)	Ly6a Ly6	1	1.2085	1.378	1.1695
P01844	Ig lambda-2 chain C region	Iglc2	1	1.2072	2.441	1.087
G3X8S8	MCG14499 (tRNA-splicing endonuclease subunit Sen15)	Tsen15 mCG_14499	1	1.2065	1.143	1.4907
F6QQ13	Selenocysteine insertion sequence-binding protein 2-like (Fragment)	Secisbp2l	1	1.2035	1.149	1.3285
P58501	PAX3- and PAX7-binding protein 1 (PAX3/7BP) (GC-rich sequence DNA-binding factor 1)	Paxbp1 Gcfc Gcfc1	1	1.2035	1.488	1.2637
Q9JLM9	Growth factor receptor-bound protein 14 (GRB14 adapter protein)	Grb14	1	1.1975	0.601	1.1215
P59328–2	WD repeat and HMG-box DNA-binding protein 1 (Acidic nucleoplasmic DNA-binding protein 1) (And-1)	Wdhd1 And1	1	1.1922	1.221	1.4022
A2A5Z6–2	E3 ubiquitin-protein ligase SMURF2 (EC 6.3.2.-) (SMAD ubiquitination regulatory factor 2) (SMAD-specific E3 ubiquitin-protein ligase 2)	Smurf2	1	1.1902	1.098	1.3955
Q8CIA9	Hippocampus abundant transcript-like protein 1	Hiatl1	1	1.1852	1.098	1.3277
H3BKB9	Protein zwilch homolog (Fragment)	Zwilch	1	1.1817	1.114	1.3972
Q5SUA5	Unconventional myosin-Ig	Myo1g	1	1.1747	1.196	1.3717
P03991	H-2 class I histocompatibility antigen, K-W28 alpha chain	H2-K1 H2-K	1	1.1682	1.554	0.966
Q61542	StAR-related lipid transfer protein 3 (Protein ES 64) (Protein MLN 64) (START domain-containing protein 3) (StARD3)	Stard3 Es64 Mln64	1	1.1672	1.663	1.496
A8C756	Thyroid adenoma-associated protein homolog	Thada Kiaa1767	1	1.165	1.299	1.382
Q80ZI6	E3 ubiquitin-protein ligase LRSAM1 (EC 6.3.2.-) (Leucine-rich repeat and sterile alpha motif-containing protein 1) (Tsg101-associated ligase)	Lrsam1	1	1.1627	1.094	1.59
F6RR81	Protein cordon-bleu (Fragment)	Cobl	1	1.1585	1.355	1.1932
Q8R2S8	CD177 antigen (CD antigen CD177)	Cd177	1	1.158	1.426	1.0102
A2ALA0	Surfeit locus protein 6	Surf6	1	1.1567	1.218	1.3962
Q5SUW0	Growth factor receptor-bound protein 10 (Fragment)	Grb10	1	1.1552	1.019	1.3747
Q9CQS2	H/ACA ribonucleoprotein complex subunit 3 (Nucleolar protein 10) (Nucleolar protein family A member 3) (snoRNP protein NOP10)	Nop10 Nola3	1	1.1455	1.37	1.1737
D3YUW8	Pogo transposable element with ZNF domain	Pogz	1	1.1365	1.373	1.3605
Q62293	Interferon-gamma-inducible GTPase Ifggb5 protein	Tgtp	1	1.1357	1.958	1.0067
Q8BX57–3	PX domain-containing protein kinase-like protein (Modulator of Na,K-ATPase) (MONaKA)	Pxk	1	1.1355	0.867	1.3607
E0CYU9	Sjoegren syndrome/scleroderma autoantigen 1 homolog	Sssca1	1	1.135	1.705	1.5802
Q9R0X0–3	Mediator of RNA polymerase II transcription subunit 20 (Mediator complex subunit 20) (TRF-proximal protein homolog)	Med20 Trfp	1	1.1335	1.088	1.3255
P18527	Ig heavy chain V region 914	0	1	1.133	1.071	0.618
A2A6A1	G patch domain-containing protein 8	Gpatch8 Gpatc8 Kiaa0553	1	1.1295	1.861	1.0447
O35242	Protein FAN (Factor associated with neutral sphingomyelinase activation) (Factor associated with N-SMase activation)	Nsmaf Fan	1	1.1275	1.116	1.417
P04184	Thymidine kinase, cytosolic (EC 2.7.1.21)	Tk1 Tk-1	1	1.1222	1.288	1.638
Q80VC9–2	Calmodulin-regulated spectrin-associated protein 3 (Protein Nezha)	Camsap3 Kiaa1543	1	1.1107	1.308	1.492
S4R2K0	Protein Pdf	Pdf	1	1.1082	1.644	1.4732
Q8BZR9	Uncharacterized protein C17orf85 homolog	0	1	1.108	1.132	1.6712
Q8K4Q0–5	Regulatory-associated protein of mTOR (Raptor) (p150 target of rapamycin (TOR)-scaffold protein)	Rptor Raptor	1	1.105	1.153	1.4442
Q6P9L6	Kinesin-like protein KIF15 (Kinesin-like protein 2) (Kinesin-like protein 7)	Kif15 Klp2 Knsl7	1	1.1012	1.367	1.3967
Q9CR76	Transmembrane protein 186	Tmem186	1	1.0997	0.655	1.0117
Q924Z6–2	Exportin-6 (Exp6) (Ran-binding protein 20)	Xpo6 Ranbp20	1	1.0997	1.209	1.5217
Q8BZT5	Leucine-rich repeat-containing protein 19	Lrrc19	1	1.0952	1.379	1.2207
P11247	Myeloperoxidase (MPO) (EC 1.11.2.2) [Cleaved into: Myeloperoxidase light chain; Myeloperoxidase heavy chain]	Mpo	1	1.0945	1.195	1.415
A8DUK4	Beta-globin (Protein Hbb-bs) (Protein Hbb-bt)	Hbbt1 Hbb-bs Hbb-bt Hbbt2	1	1.0942	2.14	0.8445
P01630	Ig kappa chain V-II region 7S34.1	0	1	1.094	1.405	1.2252
Q8CGN5	Perilipin-1 (Lipid droplet-associated protein) (Perilipin A)	Plin1 Peri Plin	1	1.0895	0.885	1.3687
Q9CQT2	RNA-binding protein 7 (RNA-binding motif protein 7)	Rbm7	1	1.0877	1.142	1.333
F7BJK1	Protein Pcdh1 (Fragment)	Pcdh1	1	1.0875	0.927	1.8367
Q80TA6–2	Myotubularin-related protein 12	Mtmr12 Kiaa1682	1	1.0835	1.091	1.5237
P54754	Ephrin type-B receptor 3 (EC 2.7.10.1) (Developmental kinase 5) (mDK-5) (Tyrosine-protein kinase receptor SEK-4)	Ephb3 Etk2 Mdk5 Sek4	1	1.082	1.341	1.1597
D3Z769	Protein lin-37 homolog (Fragment)	Lin37	1	1.0795	1.116	1.5232
A0A075B5X9	Ig heavy chain V region B1–8/186–2 (Fragment)	Ighv1–72	1	1.0795	1.415	1.2
F6TLB0	DNA-directed RNA polymerase, mitochondrial (Fragment)	Polrmt	1	1.077	1.111	1.3495
A0A087WRI5	Adenylate kinase isoenzyme 6	Ak6	1	1.075	1.346	1.1457
Q8BK35	MCG2065, isoform CRA_c (PreS1 binding protein) (Protein Gltscr2)	Gltscr2 mCG_2065	1	1.074	0.953	1.557
Q9CQT0	tRNA(His) guanylyltransferase (EC 2.7.7.79) (tRNA-histidine guanylyltransferase)	Thg1l mCG_22296	1	1.0722	1.14	1.4462
A0A075B677	Protein Igkv4–53	Igkv4–53	1	1.0705	1.361	0.986
G3UWZ0	Bromodomain adjacent to zinc finger domain protein 1A	Baz1a	1	1.0702	1.564	1.3232
F6R2G3	Mucin-4 (Fragment)	Muc4	1	1.0695	1.286	1.3432
Q6GU68	Immunoglobulin superfamily containing leucine-rich repeat protein	Islr	1	1.068	1.154	1.3577
E9PWH6	HEAT repeat-containing protein 3	Heatr3	1	1.0605	1.102	1.4625
Q8BLH7	HIRA-interacting protein 3	Hirip3	1	1.0587	1.496	1.4912
Q62264	Thyroid hormone-inducible hepatic protein (Spot 14 protein) (S14) (SPOT14)	Thrsp S14	1	1.0582	0.943	1.4015
Q99M73	Keratin, type II cuticular Hb4 (65 kDa type II keratin) (Keratin-84) (K84) (Type II hair keratin Hb4) (Type-II keratin Kb24)	Krt84 Krt2–16 Krthb4	1	1.0557	1.17	1.3252
Q9D856	Zinc transporter ZIP5 (Solute carrier family 39 member 5) (Zrt- and Irt-like protein 5) (ZIP-5)	Slc39a5 Zip5	1	1.0555	1.484	1.4005
F7BJB9	Protein Morc3	Morc3	1	1.0525	1.375	1.2087
B7ZWM8	Leucine-rich repeat and calponin homology domain-containing protein 3 (Lrch3 protein)	Lrch3	1	1.0505	1.128	1.3452
D3Z6K8	Ras-specific guanine nucleotide-releasing factor 2	Rasgrf2	1	1.0482	1.047	1.3875
Q5FWI3	Transmembrane protein 2	Tmem2 Kiaa1412	1	1.0462	1.163	1.4667
G3UZL2	RCC1 and BTB domain-containing protein 1 (Fragment)	Rcbtb1	1	1.0417	1.433	1.2967
Q61666–4	Protein HIRA (TUP1-like enhancer of split protein 1)	Hira Tuple1	1	1.0405	1.127	1.3862
P53569	CCAAT/enhancer-binding protein zeta (CCAAT-box-binding transcription factor) (CBF) (CCAAT-binding factor)	Cebpz Cbf2 Cebpa-rs1	1	1.0367	1.325	1.7395
Q9JJF3	Bifunctional lysine-specific demethylase and histidyl-hydroxylase NO66 (EC 1.14.11.-) (EC 1.14.11.27) (Histone lysine demethylase NO66)	No66 Mapjd MNCb-7109	1	1.0337	1.68	1.1642
Q9DAA6	Exosome complex component CSL4 (Exosome component 1)	Exosc1 Csl4	1	1.033	1.326	1.2455
A0A087WQR9	NEDD8-conjugating enzyme UBE2F (Fragment)	Ube2f	1	1.0292	1.352	1.2977
Q9Z0E6	Interferon-induced guanylate-binding protein 2 (GTP-binding protein 2) (GBP-2) (mGBP-2) (mGBP2) (Guanine nucleotide-binding protein 2)	Gbp2	1	1.0292	1.432	0.9467
B7ZMP1–2	Probable Xaa-Pro aminopeptidase 3 (X-Pro aminopeptidase 3) (EC 3.4.11.9) (Aminopeptidase P3) (APP3)	Xpnpep3	1	1.0285	0.927	1.4122
D3YWR2	B-cell linker protein	Blnk	1	1.0237	1.595	1.1195
H7BX32	Nuclear envelope pore membrane protein POM 121	Pom121	1	1.0165	1.187	1.3717
Q99N87	28S ribosomal protein S5, mitochondrial (MRP-S5) (S5mt)	Mrps5	1	1.0147	1.41	0.9545
Q8CBC4	Consortin	Cnst	1	1.0092	1.33	1.19
A2AER8	Polyglutamine-binding protein 1	Pqbp1	1	1.0077	1.477	0.9262
A8Y5N4	17-beta-hydroxysteroid dehydrogenase 13	Hsd17b13	1	1.006	0.603	0.7325
Q9D8I1	Marginal zone B- and B1-cell-specific protein	Mzb1 Pacap	1	1.006	1.348	0.9347
P26618	Platelet-derived growth factor receptor alpha	Pdgfra	1	1.0032	1.152	1.4392
P55088–2	Aquaporin-4 (AQP-4)	Aqp4	1	1.0005	1.327	0.8442

*Acc no* Accession number, *Prot name* Protein name, *SE* Semen Euphorbiae, *SEP* Semen Euphorbiae Pulveratum, *EFL*_*1*_ Euphorbiae Factor 1

### GO ontology analysis

**Fig. 2:**
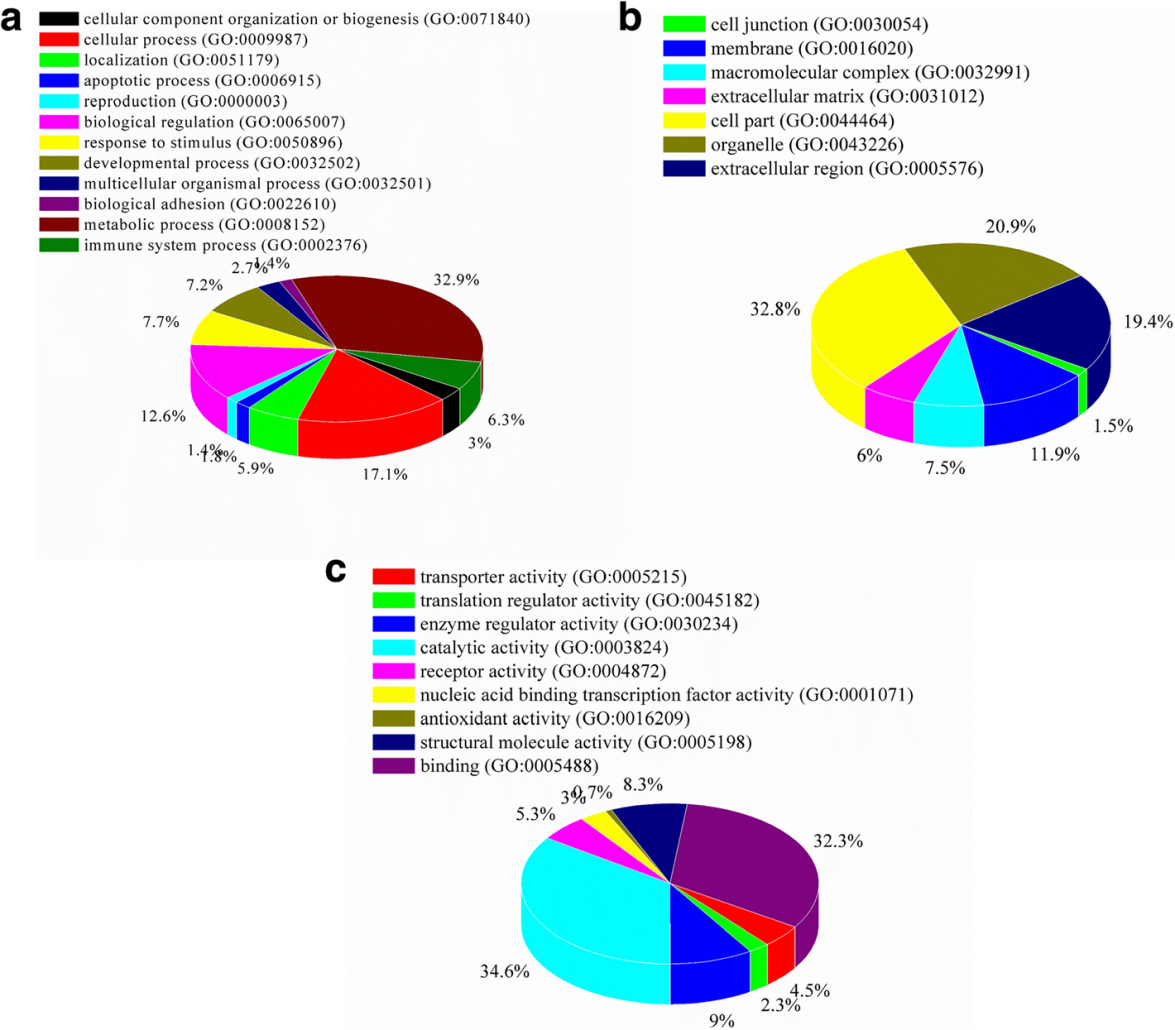
Bioinformatics analysis of the differentially expressed proteins (ratio ≥ 1.32 or ≤ 0.68 fold). **a** Biological process (**b**) Cellular component; (**c**). Molecular function

To elucidate the biological significance of these differentially modified proteins, we performed GO analysis and categorized these proteins according to their molecular function and biological process using the GO database. 295 union proteins were selected and separated into 3 categories: biological processes (Fig. 2a), cellular component association (Fig. 2b), and molecular function (Fig. 2c).

In the biological process category, the results suggested that most of the DEPs participate in metabolic processes (32.9%), cellular processes (17.10%), biological regulation (12.6%), and response to stimulus (7.70%). In the cellular component analysis, most of the potential biomarkers are concentrated in the cell part (32.80%), organelle (20.90%), extracellular region (19.40%), membrane (11.90%) or macromolecular complex. In the molecular function analysis, the differentially expressed proteins were found to play a role in catalytic activity (34.60%), binding (32.30%), enzymatic activity (9.00%) and structural molecule activity (8.30%),suggesting that their related functions were important in the colon of mice.

On the basis of our findings, it could be concluded that the identified DEPs causing by SE, SEP and EFL_1_ were mainly associated with the cellular part. The expression sites of them located within cells and organelles. G protein and Eph/Ephrin signal pathway were controlled jointly by SE and SEP. After processing, the extracts of SEP were mainly reflected in the process of cytoskeleton, glycolysis and gluconeogenesis.

### Pathway enrichment analysis and interaction network analysis

MetaCore™ (version 6.18) is an integrated software suite for functional analysis of experimental data. Differential pathways among SE, SEP, EFL_1_ and control were conducted according to the *P* Value (*P* < 0.05). All the differential pathways were shown in Tables 3, 4 and 5.

**Table 3:** Pathway Enrichment analysis of differentially expressed proteins relative to SE compared with control group

NO	Maps	pValue
1	Immune response_Oncostatin M signaling via JAK-Stat in mouse cells	0.000195
2	Immune response_Oncostatin M signaling via JAK-Stat in human cells	0.000242
3	Development_Thrombopoetin signaling via JAK-STAT pathway	0.000294
4	Immune response_IL-15 signaling via JAK-STAT cascade	0.000322
5	Development_Transcription factors in segregation of hepatocytic lineage	0.000552
6	Immune response_IL-7 signaling in T lymphocytes	0.000887
7	Immune response_IL-7 signaling in B lymphocytes	0.001136
8	Cell adhesion_Ephrin signaling	0.001244
9	Neurophysiological process_Receptor-mediated axon growth repulsion	0.001244
10	Immune response_IL-5 signaling	0.001300
11	Signal transduction_PTMs in IL-12 signaling pathway	0.001415
12	G-protein signaling_Rap1B regulation pathway	0.013047
13	Protein folding_Membrane trafficking and signal transduction of G-alpha (i) heterotrimeric G-protein	0.022438
14	Immune response_IL-12 signaling pathway	0.027103
15	Development_Glucocorticoid receptor signaling	0.028266

**Table 4:** Pathway Enrichment analysis of differentially expressed proteins relative to SEP compared with control group

NO	Maps	pValue
1	Cytoskeleton remodeling_Role of PDGFs in cell migration	0.002188
2	Glycolysis and gluconeogenesis p.3 / Human version	0.002188
3	Glycolysis and gluconeogenesis p.3	0.002188
4	Development_PDGF signaling via STATs and NF-kB	0.003877
5	Normal and pathological TGF-beta-mediated regulation of cell proliferation	0.004119
6	Cell adhesion_Ephrin signaling	0.007559
7	Neurophysiological process_Receptor-mediated axon growth repulsion	0.007559
8	Development_PDGF signaling via MAPK cascades	0.008224
9	Some pathways of EMT in cancer cells	0.009631
10	Aberrant B-Raf signaling in melanoma progression	0.011137
11	Transport_Macropinocytosis regulation by growth factors	0.014439
12	Glycolysis and gluconeogenesis (short map)	0.015773
13	G-protein signaling_Rap1B regulation pathway	0.031748
14	Cell adhesion_Chemokines and adhesion	0.034254
15	Cytoskeleton remodeling_Cytoskeleton remodeling	0.035519

**Table 5:** Pathway Enrichment analysis of differentially expressed proteins relative to EFL_1_ compared with control

NO.	Maps	pValue
1	Development_Angiopoietin - Tie2 signaling	0.000027
2	Immune response_IL-7 signaling in T lymphocytes	0.000035
3	Immune response_IL-7 signaling in B lymphocytes	0.000051
4	Immune response_Antiviral actions of Interferons	0.000090
5	Immune response_Oncostatin M signaling via JAK-Stat in mouse cells	0.000425
6	Immune response_Oncostatin M signaling via JAK-Stat in human cells	0.000526
7	Development_Thrombopoetin signaling via JAK-STAT pathway	0.000639
8	Immune response_IL-15 signaling via JAK-STAT cascade	0.000699
9	Immune response_IL-23 signaling pathway	0.000827
10	Signal transduction_PTMs in IL-23 signaling pathway	0.001274
11	Development_PDGF signaling via STATs and NF-kB	0.001357
12	Immune response_IL-22 signaling pathway	0.001532
13	Development_EPO-induced Jak-STAT pathway	0.001623
14	Development_Growth hormone signaling via STATs and PLC/IP3	0.001623
15	Immune response_IL-9 signaling pathway	0.001717

Comparing with group 1(control), the pathways with higher activity were mainly related to the immune response, and also related to other physiological processes such as development and G protein pathways; the dominant signaling pathways were interleukin signaling pathway, JAK/Stat et al.; the key proteins involved in multiple pathways contain STAT1, SERPINA3, G protein Rap1B and so on. Meanwhile, group 4 (EFL_1_) showed that the physiological process with high activity was relatively simple, mainly focused on the immune response and development process. Interleukin signaling pathways, Ang/Tie 2 and NF/kB were found to be the main signaling pathways and the key proteins involved were STAT1 and STAT5; compared with control, group 3 induced cytoskeleton remodeling, glycolysis and gluconeogenesis with higher activities, signaling pathways which contain a variety of major B-Raf pathways, epithelial cells to interstitial cell transition(EMT)-related signaling pathways, cell endocytosis, etc. and PDGF receptors, Ephrin receptors,in which STAT 1 was related to the key proteins.

**Fig. 3:**
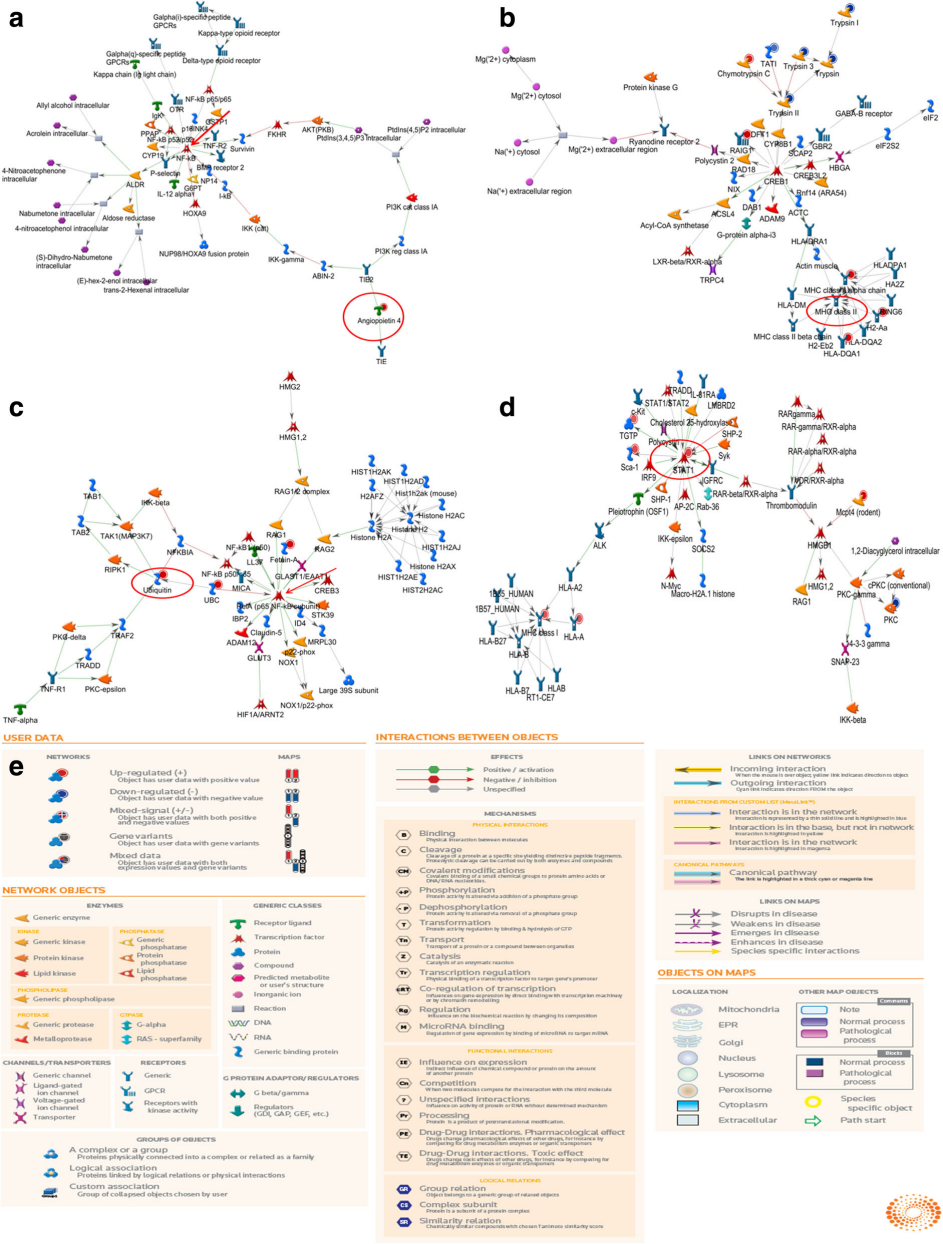
Biological networks generated by different groups. **a** Protein interaction networks of DEPs from four groups after taking the intersection; **b**, **c** and **d**: protein interaction networks of DEPs from four groups after taking union (**b**: Major Histocompatibility Complex class IInetwork; **c**: Ubiquitination in Mediating the Cellular Stress Response; **d**: Interferon-γ-mediated signal transduction and response network); **e** Explanation of various symbols in the network map. The network of significantly differentially expressed proteins (ratio ≥ 1.32 or ≤ 0.68 fold) was analyzed by MetaCoreTM(version 6.18)software

A network was constructed by protein-protein interaction of the 295 significantly DEPs basing on Analyze Network Algorithm using MetaCore in Fig. 3 (A-D). (Tables 6 and 7).

**Table 6:** Intersection of differentially expressed protein Networks

Network	GO processes	Total nodes	Seed nodes	p-Value	zScore	gScore
Angiopoietin 4, NF-kB, ALDR, TIE2, ATP + PtdIns(4,5)P2 = ADP + PtdIns(3,4,5)P3	response to oxygen-containing compound (70.6%; 1.570e-16), regulation of multicellular organismal process (76.5%; 2.094e-15), response to peptide (47.1%; 1.618e-14), response to stress (82.4%; 2.570e-14), positive regulation of cellular process (88.2%; 3.104e-14)	51	1	0.00245	20.16	22.66

**Table 7:** Union of differentially expressed protein Networks

Network	GO processes	p-Value	zScore	gScore
Trypsin II, Chymotrypsin C, Trypsin 3, TATI, RAIG1	antigen processing and presentation of peptide or polysaccharide antigen via MHC class II (27.3%; 6.498e-17)	1.010E-21	48.76	48.76
Ubiquitin, Fetuin-A, UBC, RelA (p65 NF-kB subunit), TRAF2	regulation of response to stress (56.5%; 6.254e-19), positive regulation of NF-kappaB transcription factor activity (28.3%; 2.556e-17)	1.140E-05	14.33	44.33
STAT1, TGTP, Mcpt4 (rodent), Sca-1, Thrombomodulin	interferon-gamma-mediated signaling pathway (31.9%; 8.694e-24), response to interferon-gamma (36.2%; 1.175e-23)	2.970E-14	33.37	33.37

Obviously, commonly pathways are mainly interleukin-mediated signaling pathways, including IL-7, IL-15, IL-23 and other inflammatory factors both controlled by EFL_1_ and SE groups. We supposed that these inflammatory factors activate the interleukin signaling pathway, NF / kB signaling pathway, and then mediate intestinal mucosal barrier injure by up-regulating inflammatory proteins expression which resulting in inflammatory response. While there is no obvious interleukin-mediated inflammatory response in SEP group. Generally speaking, inflammatory response especially interleukin might be closely related to the attenuated mechanism of Semen Euphorbiae.

According to network analysis, four reliable functional networks were found and analyzed. After intersection of four groups, the main protein interaction network was multicellular organism regulation process (only Angiopoietin 4 is the down-regulated differentially expressed protein and NF-κB is a pivotal role which interacts with other proteins in the network most closely, Fig. 3a). DEPs which were taken together mainly participated in the protein interaction networks as shown in Fig. 3b, c and d. MHC II presents endogenous and exogenous antigenic peptides or antigenic polysaccharides (containing 10 differential proteins, the key point is MHC class II in Fig. 3b), stress response (containing 3 up-regulation differential proteins, RelA/P65 and ubiquitin are the central part of network, Fig. 3c), γ- Interferon - mediated signal transduction and response (containing 6 up-regulation,1 down-regulation differential proteins, as shown in Fig. 3d, STAT1 interacted closely with other proteins and play an important role in the networks).

It should be pointed out that Angiopoietin 4 is the only down-regulated differential expressed protein in the interaction network. Subsequently, STAT1 was found to be the key protein shared by the EFL_1_, SEP and SE tested groups, compared with the control group. A previous study has implied that the transcription factor NF-κB (nuclear factor kappa B) plays a central role in the regulation of immune and inflammatory responses, as well as in control of cell apoptosis. These proteins participate in the regulation of a wide range of genes involved in immune, inflammatory and apoptosis function [21]. Although the relationship between Angiopoietin 4 and NF-κB has not been reported, according to the network, we could make the hypothesis that SE could increase Angiopoietin 4 and then activate NF-κB to make the body produce immune or inflammatory response. In addition, interferons (IFNs) are important cytokines that play essential roles in antiviral, antibacterial, antitumor and immunomodulatory activities. IFNs primarily signals through the JAK-STAT pathway leading to the activation of signal transducer and activator of STAT and subsequent transcription of target genes [22]. Based on the pathway analysis, IFN-γ could activate STATs through JAK-STAT signal pathway to initiate CIITA (typeIItranscription activator) which as MHC IItrans activator, and then the expression of MHC II were up-regulated to produce immune response and immune regulation so that the mice have diarrhea symptoms after treated with SE group. For these reasons and hypothesis, western blot analysis was then conducted to validate the two differentially expressed proteins- STAT1 and Angiopoietin 4.

### Validation of differentially expressed proteins identified by proteomics

**Fig. 4:**
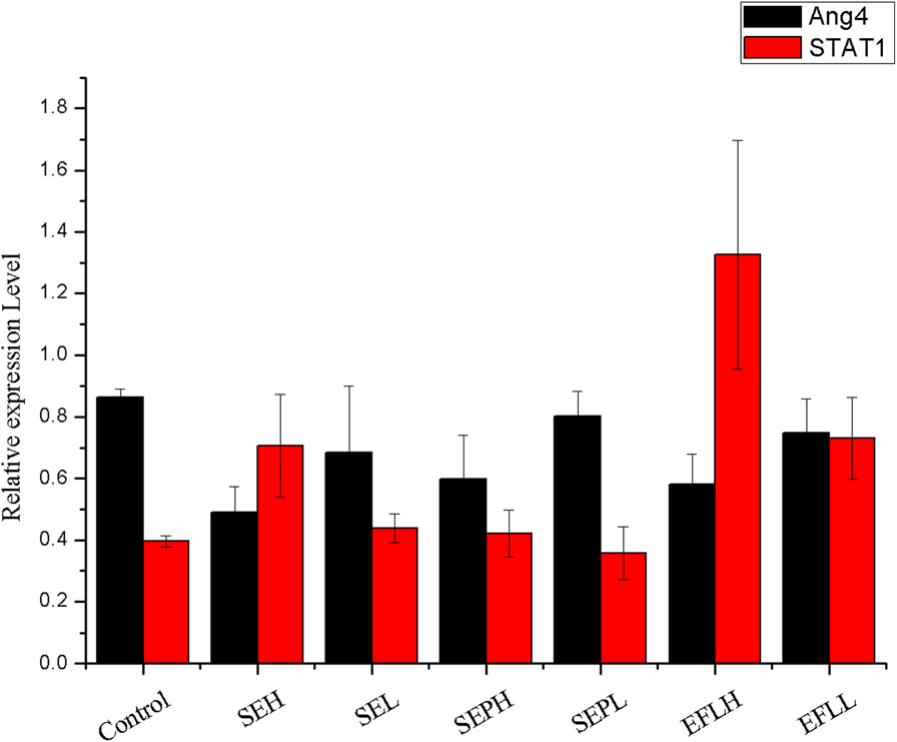
Relative expression levels of Ang4 and STAT1were normalized to the β-actin which were quantified by densitometric analysis. These experiments were each conducted five times

**Fig. 5:**
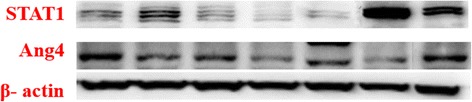
Western blotting showing the changes in Ang4 and STAT1 level in mice intestine treated with different doses of SE, SEP and EFL_1_ with respect to control-treated mice intestine Note:Internal reference:β- actin,1.Control, 2.High-dose of SE (SEH*,* 1.5 ml/20 g), 3.low-dose of SE (SEL*,* 0.5 ml/20 g), 4. High-dose of SEP (SEPH*,* 1.0 ml/20 g), 5. Low-dose of SEP (SEPL*,* 0.33 ml/20 g), 6.High-dose of EFL_1_ (EFLH*,* 20 mg/20 g), 7 Low-dose of EFL_1_ (EFLL*,* 10 mg/20 g)

Two proteins, STAT1 and Angiopoietin 4 identified DEPs with marked differences in expression determined by iTRAQ based quantitative analysis were selected to be verified by western blot analysis (Figs. 4 and 5). As depicted in Figs. 4 and 5 and Table 8, Angiopoietin 4 protein was significantly down-regulated in SEH, SEPH and EFLH groups as compared with control group (*p* < 0.05), the expression level of Ang4 in SEH was the lowest; and STAT1 was up-regulated in SEH, SEPH and EFLH groups, which levels were all higher than control group (*p* < 0.05). Moreover, the groups of low dose of SEL, SEPL and EFLL have no significant differences compared with the control. The results which were found by western blot is consistent with the findings in iTRAQ analysis. Both of Ang-4 and STAT1 expression levels in the mice colon tissue may be dose-dependent with the increase dose of SE and SEP.

**Table 8:** The relative expression of Ang4 and STAT1 in intestinal tissue of mice ($\overline{X}$±S, *n* = 5)

groups	Ang4	STAT1
Control	0.865 ± 0.027	0.396 ± 0.019
SEH	0.489 ± 0.084*	0.706 ± 0.167*
SEL	0.683 ± 0.218	0.439 ± 0.046
SEPH	0.598 ± 0.142*	0.421 ± 0.076
SEPL	0.803 ± 0.080	0.358 ± 0.086
EFLH	0.582 ± 0.098*	1.326 ± 0.372*
EFLL	0.749 ± 0.111	0.731 ± 0.133

Note: *compared with control (*P* < 0.05)

It is well established that the angiopoietin (Ang) family of growth factors includes Ang1, Ang2, Ang3 and Ang4, all of which bind to the endothelial receptor tyrosine kinase Tie2. Ang3 (mouse) and Ang4 (human) are interspecies orthologs. Tie2 [23] maintains the vascular integrity of mature vessels by enhancing endothelial barrier function and inhibiting apoptosis of endothelial cells. According to the pathway network analysis, as shown in Fig. 3a, we speculated that Semen Euphorbiae might inhibit the expression of Ang-4, which Tie-2 couldn’t be activated, so that the steady state of endothelial cells was broken and the sensitivity of various inflammatory mediators increased, permeability, and thus promoted the occurrence of inflammatory response. The inhibition of Ang 4 by SEP group after processing was weakened comparing to SE group, resulting in lower diarrhea and inflammatory response.

**STAT1** has been implicated as a mediator of biological responses to a variety of growth factors and cytokines, based on ligand-dependent tyrosine phosphorylation and activation. Stat1 is a functional transcription factor even in the absence of inducer-mediated activation, participating in the constitutive expression of some genes [24]. JAK2/ STAT pathway signaling is activated by a wide array of cytokines and growth factors leading to the stimulation of cell proliferation, differentiation, and apoptosis [25]. And it is an important way of signal transduction of inflammatory factors.

In addition to being involved in the main JAK2 / STAT signaling pathway, STAT1 could be activated by JAK2 (non-receptor tyrosine) kinase, but also by inflammatory factors such as interleukin-6 (IL-6), tumor necrosis factor (TNF),growth factors such as interferon (IFN) [26], epidermal growth factor (EGF), platelet-derived growth factor (PDGF) and other signal activation.

As the up-regulated proteins induced by each group, STAT1 was induced by SEP group lower than the SE group so that we suspected that STAT 1 was most likely one of target proteins related to intestinal inflammation which might illustrate the attenuated mechanism of Semen Euphorbiae.

Both Ang-4 and STAT1 were surmised to be one of the target proteins inducing by Semen Euphorbiae.

## Conclusions

This study used iTRAQ labeling followed by 2D-LC-MS/MS for the quantitative proteomic analysis of intestine samples from KM mice with different groups and control to discover candidate biomarkers for attenuated mechanism of Semen Euphorbiae processing for the first time. These findings suggest that SE induced an inflammatory response, and activated the Interleukin signaling pathway, such as the Ang/Tie 2 and JAK2/ STAT signaling pathways, which may eventually contribute to injury result from intestinal inflammatory, while SEP could ease this injury by reducing STAT1 and activating Ang-4 which could reduce the inflammatory response. Taken together, these results not only provided a novel insight into attenuated mechanism of Semen Euphorbiae, which was marked by a number of DEPs that might be associated with intestinal inflammation, but also the first experimental evidence that the Angiopoietin 4 and STAT1 proteins might be two major candidate biomarkers in the attenuated of SE after processing based on proteomic investigation. Our findings suggest that this screening method has potential valuable in studying mechanism of processing. Future systematic studies will investigate how Semen Euphorbiae regulate the expression of these key proteins and illustrate the problem from a clinical point of view.

## Abbreviations

2D-LC-MS/MS: Two-dimensional liquid chromatography-tandem mass spectrometry
ACN: Acetonitrile
Ang: Angiopoietin
CIITA: TypeIItranscription activator
DEPs: Differentially expressed proteins
DTT: Dithiothreitol
EFL_1_: Euphorbiae Factor 1
EGF: Epidermal growth factor
Eph/Ephrin: Erythropoientin-producing hepatocyte kinases/Eph receptor interacting proteins
GO: Gene ontology
IFN: Interferon
IgG: Immunoglobulin G
IL-6: Interleukin-6
iTRAQ: Isobaric tags for relative and absolute quantitation
JAK2: Janus Kinase 2
LC: Liquid chromatography
NF-κB: Nuclear factor kappa B
PDGF: Platelet-derived growth factor
PSMs: Peptide-spectrum matches
PVDF: Polyvinylidene fluoride
SDS-PAGE: Sodium dodecyl sulfate polyacrylamide gel electrophoresis
SE: Semen Euphorbiae
SEP: Semen Euphorbiae Pulveratum
STAT1: Signal transducers and activators of transcription one
TCM: Traditional Chinese medicine
TNF: Tumor necrosis factor
